# Single-leg versus double-leg weight-bearing radiographs for coronal alignment assessment in patients with lower extremity trauma

**DOI:** 10.1051/sicotj/2026073

**Published:** 2026-10-12

**Authors:** Naya Pabla, Kairo Noronha, Sathyagopal Siddapureddy, Tachelle Ting, Joseph Cavataio, Elliot Majlessi, Curtis Wilkerson, Rahul Vaidya, Robert Teitge

**Affiliations:** 1 Wayne State University School of Medicine and Detroit Medical Center Detroit MI USA; 2 Flinders University Adelaide SA 5042 Australia; 3 Michigan State University College of Osteopathic Medicine East Lansing MI USA

**Keywords:** Lower extremity alignment, Weight-bearing radiographs, Coronal alignment, Osteotomy, Deformity correction, Postoperative imaging

## Abstract

*Introduction*: Accurate assessment of coronal lower extremity alignment is critical for surgical planning and postoperative evaluation following orthopedic trauma. Although double-leg (DL) standing radiographs remain the standard for alignment assessment, single-leg (SL) stance more closely approximates physiologic loading during gait. Prior studies have demonstrated differences between DL and SL alignment measurements in patients with osteoarthritis and elective deformity correction, but comparable data in orthopedic trauma patients are lacking. *Methods*: We performed a retrospective study of patients presenting to a Level I trauma center after lower extremity orthopedic surgery who underwent DL and SL standing radiographs during the same clinic visit. Lateral distal femoral angle, medial proximal tibial angle, and joint line convergence angle were measured by two blinded evaluators and averaged. The primary outcome was the proportion of limbs with at least one SL–DL difference exceeding the prespecified threshold (>3° for LDFA or MPTA; >2° for JLCA). Bland–Altman analyses assessed agreement, with patient-cluster bootstrap confidence intervals accounting for patients who contributed multiple limb encounters. *Results*: Forty-nine encounters from 32 patients contributed 91 limb-encounters (64 operated and 27 non-operated). Overall, 69.2% were within all prespecified thresholds, whereas 30.8% exceeded at least one threshold. The proportion exceeding at least one threshold was 40.6% in operated limbs and 7.4% in non-operated limbs. Bland–Altman biases were 0.00° for LDFA, −0.06° for MPTA, and 0.13° for JLCA; corresponding 95% limits of agreement were −5.46° to 5.46°, −4.26° to 4.15°, and −2.74° to 3.00°, respectively. *Discussion*: Average DL–SL differences were small, but nearly one-third of limbs exceeded at least one prespecified descriptive threshold. Because this study did not establish the accuracy of either imaging condition against an external reference standard, SL radiographs should be viewed as potentially complementary to, rather than superior to, DL radiographs in selected postoperative patients.

## Introduction

Lower-extremity alignment assessment is fundamental to surgical planning and postoperative evaluation in orthopedic trauma. Accurate measurement of coronal alignment, including the lateral distal femoral angle (LDFA), medial proximal tibial angle (MPTA), and joint line convergence angle (JLCA), guides deformity correction, implant positioning, and assessment of surgical outcomes. Accurate restoration of the mechanical axis is critical because coronal alignment influences load distribution across the lower extremity, implant longevity, and functional outcomes, making precise radiographic assessment essential for preoperative planning and postoperative evaluation [1]. Standardized radiographic measurement techniques are also essential to ensure reproducible assessment of lower extremity alignment across patients and studies [2].

Double-leg (DL) standing radiographs are the current standard for evaluating lower extremity alignment. However, the single-leg (SL) stance more closely approximates physiologic loading because SL support accounts for approximately 60% of the gait cycle [3]. During DL stance, load is distributed across both limbs, potentially reducing joint space narrowing and altering coronal alignment measurements compared with SL loading [3, 4].

Previous investigations comparing DL and SL radiographs have primarily evaluated patients with knee osteoarthritis or healthy volunteers. A recent systematic review by Colyn et al. reported a mean difference of 1.43° in hip-knee-ankle angle between DL and SL stances and concluded that reliance on DL radiographs may underestimate coronal deformity [5]. Similarly, Sabzevari et al. demonstrated increased varus alignment during SL stance in patients undergoing high tibial osteotomy, with much of the change attributable to differences in JLCA [6]. Together, these studies suggest that weight-bearing conditions can influence coronal alignment measurements and may affect surgical decision-making [7].

Whether these findings apply to patients following orthopedic trauma remains uncertain. Unlike elective reconstructive populations, trauma patients often have altered joint mechanics, retained implants, postoperative pain, and compensatory loading patterns that may further influence alignment under physiologic weight-bearing conditions. Despite the widespread use of long-leg standing radiographs in trauma practice, agreement between DL and SL imaging in this population has not been well characterized.

The purpose of this study was to evaluate agreement between DL and SL weight-bearing radiographs for coronal alignment assessment in patients following lower extremity orthopedic surgery. We hypothesized that a clinically important proportion of limbs would demonstrate alignment discrepancies between the two imaging conditions, particularly in operated limbs.

## Materials and methods

This retrospective study included patients aged 18 years or older who presented to a Level I trauma center after lower extremity orthopedic surgery. No upper age limit was applied. Inclusion required interpretable SL and DL full-length standing radiographs obtained during the same clinic visit and available in PACS. Ability to complete both standing acquisitions was the practical weight-bearing requirement. Postoperative interval was not used as an analytic eligibility restriction. Represented indications included fractures of the femur, tibia, fibula, or patella; deformity correction; malunion; nonunion; and osteomyelitis. Initial, follow-up, bilateral, repeat-visit, operated, and contralateral non-operated limb encounters were eligible. Revision status was not a separately coded exclusion.

Seventy-four encounters were identified; 25 were excluded because a paired SL–DL radiographic set was unavailable, leaving 49 encounters. At the limb level, exclusion was limited to absence of a paired acquisition, incomplete visualization of the relevant anatomy, or image quality inadequate for measurement. No diagnosis-specific limb exclusion was applied beyond these imaging requirements. Fixed flexion deformity, genu recurvatum, posterior tibial slope, and postoperative stiffness were not measured and therefore were not used as exclusion criteria. The exact interval from surgery to imaging was not abstracted.

The final cohort comprised 49 encounters from 32 distinct patients. Injuries involved the femur (*n* = 16), tibia (*n* = 12), and patella (*n* = 1). Procedures included osteotomy or deformity correction (*n* = 15), open reduction and internal fixation (*n* = 7), intramedullary nailing (*n* = 2), external fixation (*n* = 1), nonunion or malunion repair without osteotomy (*n* = 1), and adjunct procedures including manipulation under anesthesia and irrigation and debridement (*n* = 3). The 49 encounters contributed 91 analyzable limbs: 64 operated and 27 non-operated. Thus, neither limbs nor encounters were assumed to be statistically independent observations.

Standing radiographs were obtained as part of the institution’s routine full-length lower-extremity imaging workflow. Because this was a retrospective PACS study, the source dataset did not record exact foot position, patellar orientation, degree of limb rotation, use of external support, beam settings, or quantitative load borne by the tested limb during SL acquisition. These factors could not be standardized retrospectively and were treated as potential sources of variability. Coronal alignment measurements were obtained using the Bonesetter application. The locked 91-limb dataset contained paired DL and SL values for LPFA, LDFA, MPTA, LDTA, and JLCA. LDFA, MPTA, and JLCA formed the prespecified threshold-based primary outcome because they localized distal femoral, proximal tibial, and joint-line contributions and had a priori discrepancy thresholds. Definitions and reference ranges are summarized in Table 1 [8–10]. Two blinded evaluators independently measured each radiographic parameter. The mean of their two measurements was used for analysis; however, the individual evaluator-level values were not retained after averaging. Representative pre- and post-osteotomy radiographs are shown in Figures 1 and 2, respectively.

**Table 1:** Definitions and reference values for alignment parameters measured on DL and SL full-length weight-bearing radiographs. Values and measurement conventions were adapted from Hess et al., Moreland et al., and Sabharwal et al. [8–10]. Reference values describe published populations and should not be interpreted as universal clinical targets.

Angle	Normal/Abnormal	Definition
Hip-knee-ankle angle (HKAA)	Normal is 180°. Angle >180° is a valgus deformity. Angle <180° is a varus deformity.	Measures the degree of overall deformity
Lateral proximal femoral angle (LPFA)	Normal is 85°–95°	Angle between the femoral mechanical axis and the proximal femoral joint line
Lateral distal femoral angle (LDFA)	Normal is 93°	Angle between the femoral mechanical axis and the distal femoral joint line.
Medial proximal tibial angle (MPTA)	Normal is 87°	Angle between the tibial mechanical axis and proximal tibial joint line.
Lateral distal tibial angle (LDTA)	Normal is 86°–92°	Angle between the anatomical axis of the tibia and the distal tibial articular surface
Joint line convergence angle (JLCA)	Normal is 0°–2°	Angle between the distal femoral and proximal tibial joint line. Hess et al., 2019
Leg length (in.)		Distance from center of femoral head to center of distal tibia joint surface.

**Figure 1:**
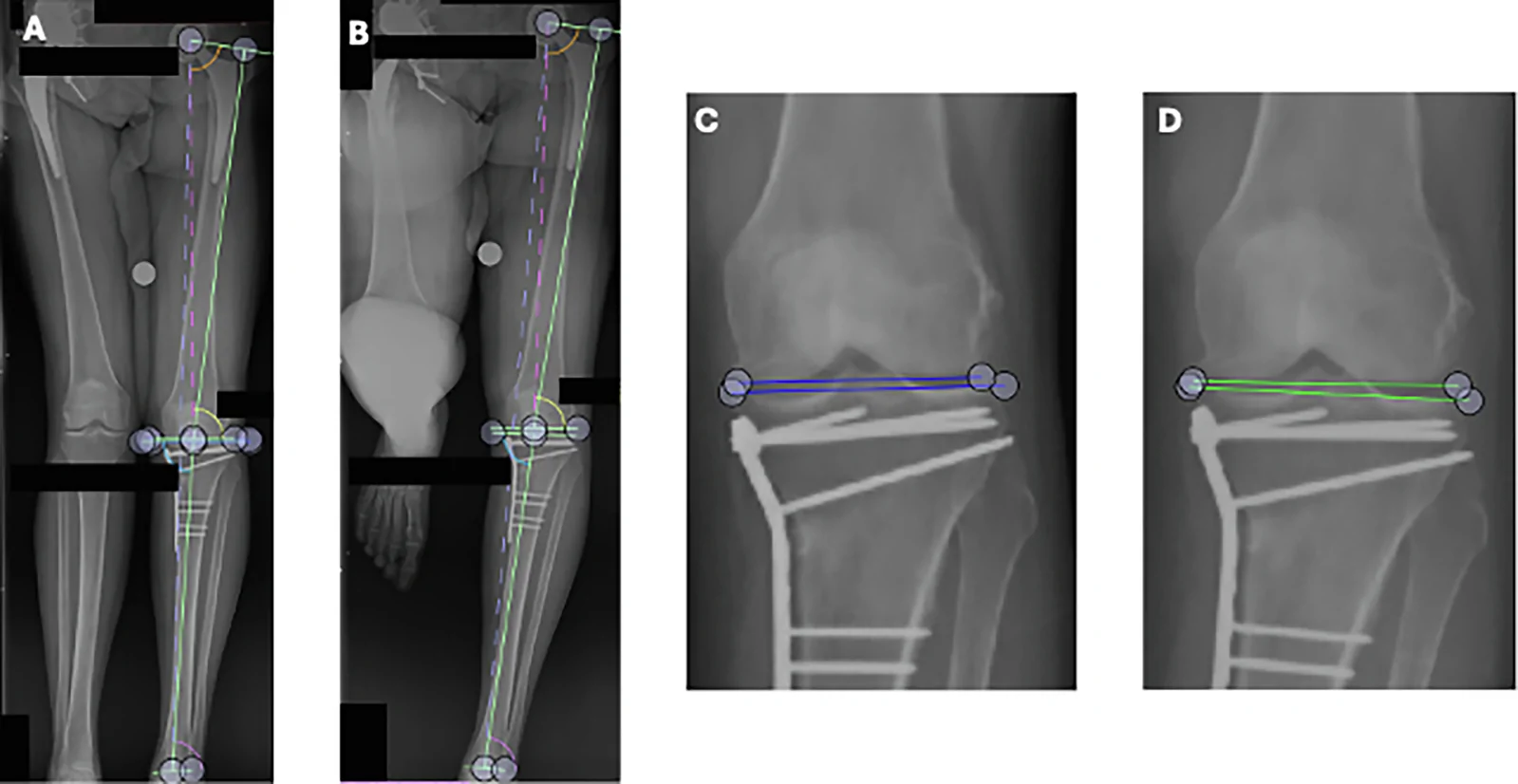
Representative pre-osteotomy full-length standing radiographs obtained under double-leg and single-leg weight-bearing conditions. (A) Double-leg standing radiograph. (B) Single-leg standing radiograph. (C) Magnified view of the knee on the double-leg standing radiograph. (D) Magnified view of the knee on the single-leg standing radiograph.

**Figure 2:**
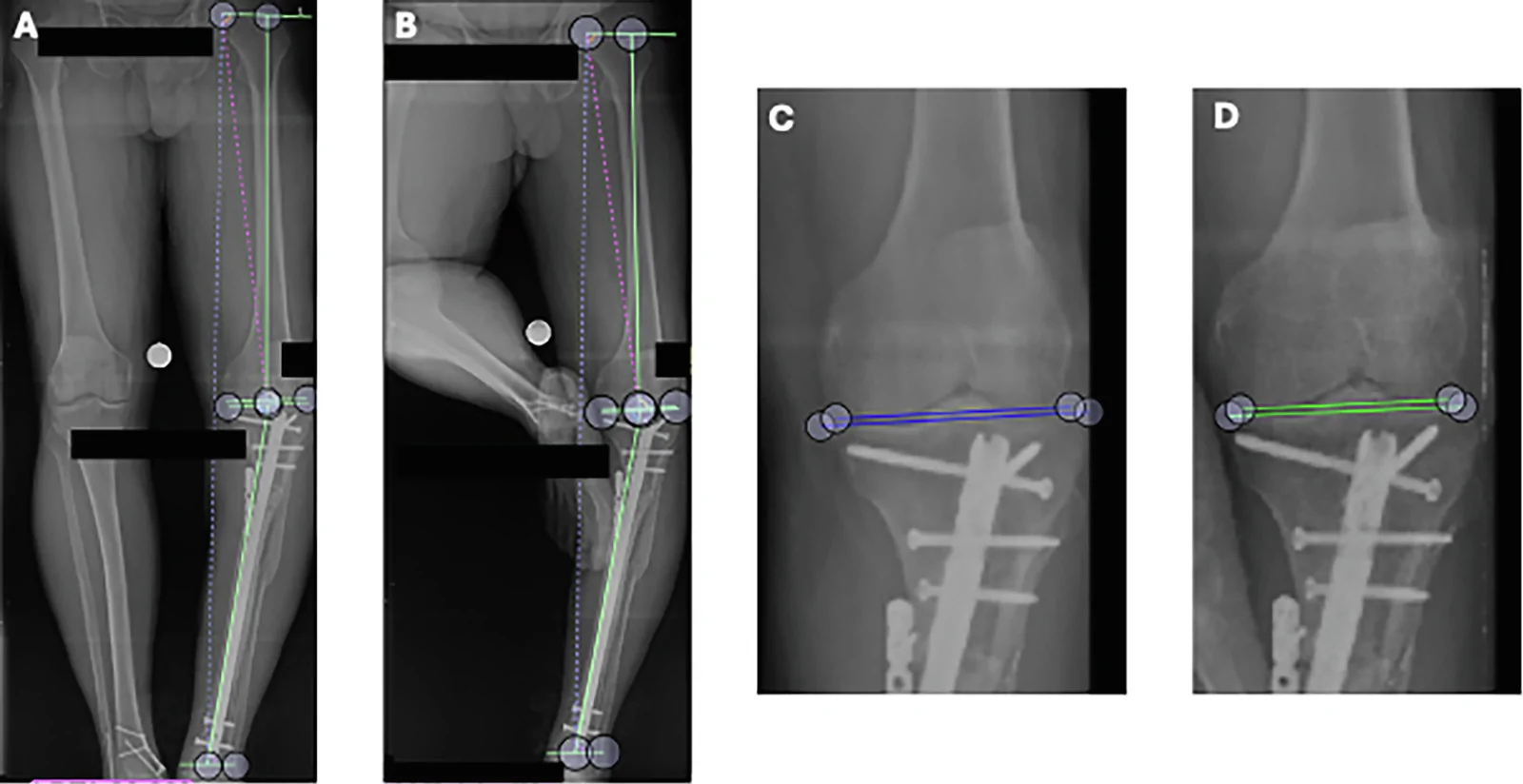
Representative post-osteotomy full-length standing radiographs obtained under double-leg and single-leg weight-bearing conditions. (A) Double-leg standing radiograph. (B) Single-leg standing radiograph. (C) Magnified view of the knee on the double-leg standing radiograph. (D) Magnified view of the knee on the single-leg standing radiograph.

For each limb, the absolute SL–DL difference was classified as exceeding the prespecified descriptive threshold when it was >3° for LDFA or MPTA or >2° for JLCA. These cut points summarized potentially relevant modality differences; they were not validated minimal clinically important differences and do not establish the accuracy of either imaging condition. The 3° threshold was informed by prior alignment literature, while the narrower 2° JLCA threshold reflected the smaller expected range of this joint-level angle [8–10]. Sensitivity analyses were conducted to recalculate LDFA and MPTA agreement using 2°, 3°, and 4° thresholds.

The primary descriptive outcome was the proportion of limbs with at least one absolute SL–DL difference exceeding the prespecified threshold for LDFA, MPTA, or JLCA. Secondary outcomes were parameter-specific mean difference (DL − SL), mean absolute difference, and within-threshold proportions, summarized overall and by operative status.

LPFA, LDFA, MPTA, LDTA, and JLCA were reported as continuous agreement measures and included in Bland–Altman analyses. LDFA, MPTA, and JLCA were included in the prespecified threshold-based primary outcome.

For LPFA, LDFA, MPTA, LDTA, and JLCA, the paired difference (DL − SL), mean absolute difference, and Bland–Altman agreement were calculated. Bias was defined as the mean of the paired differences, and 95% limits of agreement (LoA) were calculated as bias ±1.96 times the standard deviation of the paired differences [15, 16]. Bland–Altman plots display each paired difference against the mean of the DL and SL measurements. Exploratory linear regression assessed proportional bias. Because some patients contributed bilateral limbs and/or more than one visit, 95% confidence intervals for bias, LoA, threshold agreement, and the combined primary outcome were estimated using a 10,000-iteration patient-cluster bootstrap that resampled the 32 patients with replacement while retaining all observations from each sampled patient [16]. Each row represented a single radiographic limb encounter; dependence was handled at the patient level.

The Institutional Review Board approved this retrospective study of Detroit Medical Center (IRB Protocol No. IRB-21-10-4123). The requirement for informed consent was waived because the study involved retrospective review of existing clinical and radiographic data and posed minimal risk to participants.

## Results

A total of 74 encounters were screened; 25 (33.8%) were excluded because a paired SL–DL standing radiographic set was unavailable. The final cohort included 49 encounters from 32 patients and 91 limb-encounters (64 operated and 27 non-operated). Some patients contributed bilateral limbs and/or more than one visit; patient-level clustering was retained in bootstrap resampling.

Overall, 69.2% of limb-encounters (63/91; patient-cluster bootstrap 95% CI, 58.8%–79.3%) were within all prespecified thresholds, whereas 30.8% (28/91) exceeded at least one threshold. The proportions exceeding at least one threshold were 40.6% in operated limb encounters and 7.4% in non-operated limb-encounters.

Agreement for individual alignment parameters is summarized in Table 2. At the prespecified thresholds, LDFA agreement was 85.7%, MPTA agreement was 91.2%, and JLCA agreement was 86.8%. In sensitivity analyses, LDFA agreement across all 91 limb-encounters was 71.4%, 85.7%, and 92.3% at 2°, 3°, and 4°, respectively; corresponding MPTA agreement was 70.3%, 91.2%, and 92.3%. Operated limb-encounters consistently demonstrated lower agreement than non-operated limb-encounters across thresholds.

**Table 2:** The mean bias, mean absolute error, and proportion of limbs within the predefined tolerance for the LDFA, MPTA, and JLCA angles. CI: Confidence interval.

Group	Predefined tolerance	*n*	Mean bias (DL − SL)	Mean absolute error	Percent within tolerance	95% CI
*Lateral distal femoral angle (LDFA)*						
All limbs	3°	91	0.004°	1.713°	85.7%	[79.0%–92.0%]
Non-operated	3°	27	−0.431°	1.264°	92.6%	[80.0%–100.0%]
Operated	3°	64	0.187°	1.903°	82.8%	[74.2%–90.9%]
*Medial proximal tibial angle (MPTA)*						
All limbs	3°	91	−0.058°	1.591°	91.2%	[83.3%–96.9%]
Non-operated	3°	27	−0.257°	1.029°	100.0%	[100.0%–100.0%]
Operated	3°	64	0.026°	1.829°	87.5%	[76.0%–95.7%]
*Joint line convergence angle (JLCA)*						
All limbs	2°	91	0.131°	1.033°	86.8%	[78.7%–94.3%]
Non-operated	2°	27	−0.004°	0.860°	100.0%	[100.0%–100.0%]
Operated	2°	64	0.188°	1.105°	81.3%	[69.2%–91.7%]

Bland–Altman analysis included all 91 limb-encounters for LPFA, LDFA, MPTA, LDTA, and JLCA. Mean DL − SL biases were −0.92°, 0.00°, −0.06°, 0.39°, and 0.13°, respectively. Corresponding 95% LoA were −12.77° to 10.93° for LPFA, −5.46° to 5.46° for LDFA, −4.26° to 4.15° for MPTA, −5.31° to 6.09° for LDTA, and −2.74° to 3.00° for JLCA (Table 4; Figure 3). Patient-cluster bootstrap confidence intervals are reported in Table 4. No evidence of proportional bias was identified for LPFA (*p* = 0.969), LDFA (*p* = 0.502), MPTA (*p* = 0.071), or LDTA (*p* = 0.608). JLCA demonstrated evidence of proportional bias (slope = 0.36; unadjusted *p* = 0.007; patient-cluster bootstrap 95% CI for slope, 0.03–0.68), indicating that the DL − SL difference increased as mean JLCA increased. Although average biases were small, the LoA demonstrate wider individual SL–DL differences, particularly for LPFA.

**Figure 3:**
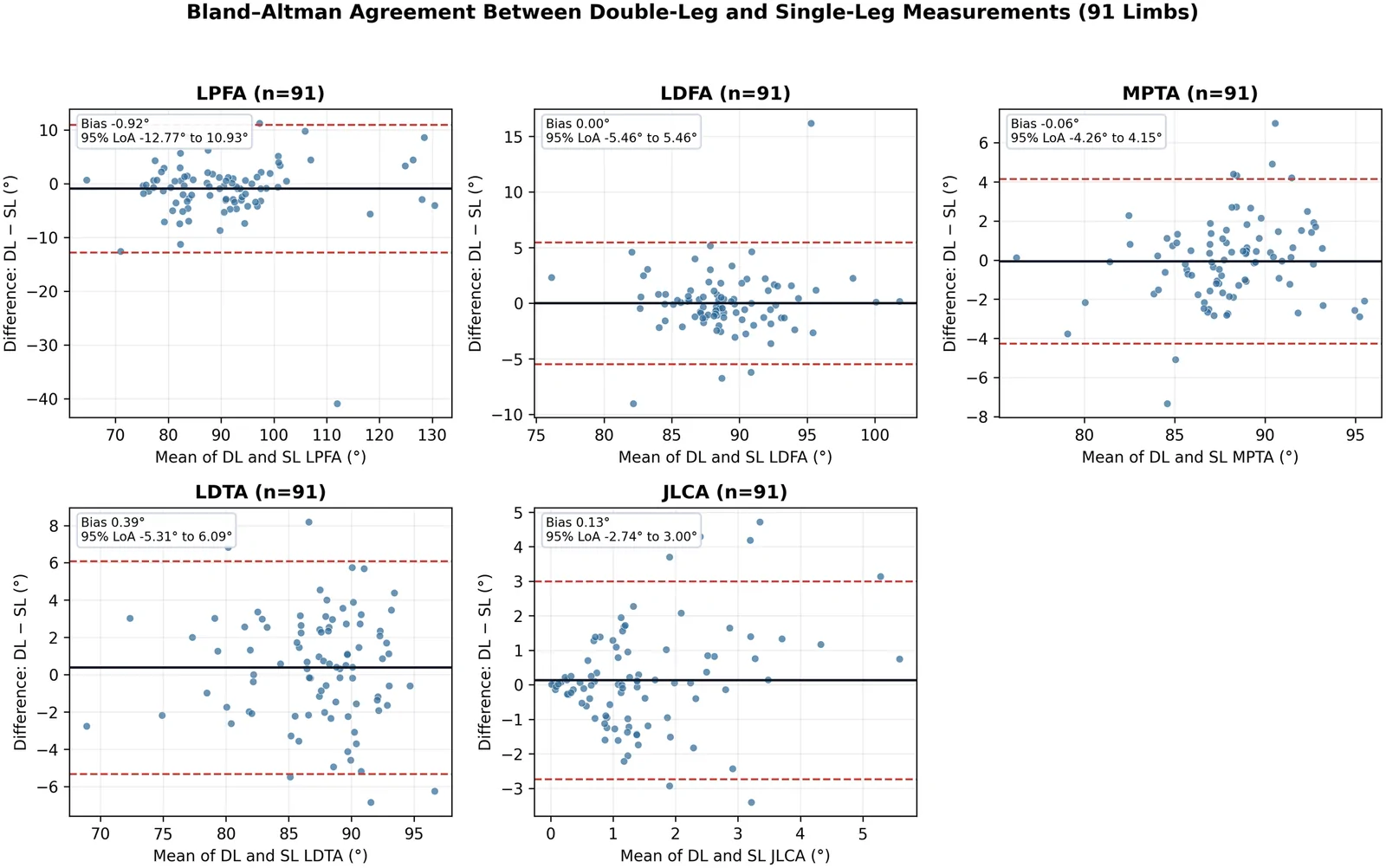
Bland–Altman plots for LPFA, LDFA, MPTA, LDTA, and JLCA in 91 limb encounters. Solid lines denote mean paired differences (DL − SL), and dashed lines denote 95% limits of agreement (bias ± 1.96 SD).

When discrepancies were present, they most commonly involved a single alignment parameter. Isolated LDFA discrepancies occurred in 11.0% of limbs, followed by isolated JLCA (8.8%) and isolated MPTA (5.5%). Multi-parameter discrepancies were uncommon, with LDFA + JLCA and MPTA + JLCA each occurring in 2.2% of limbs and LDFA + MPTA occurring in 1.1%.

Subgroup analyses of operated limbs are presented in Table 3. Fracture history was not associated with increased disagreement between DL and SL radiographs (41.4% vs. 40.0%; Fisher’s exact *p* = 1.000). Limbs with a history of osteotomy demonstrated a higher proportion of discrepant measurements than those without osteotomy (50.0% vs. 34.2%; Fisher’s exact *p* = 0.300), although this difference did not reach statistical significance. Within the osteotomy subgroup, LDFA demonstrated the lowest agreement (76.9% within tolerance), whereas MPTA remained highly reproducible (92.3% within tolerance).

**Table 3:** Subgroup analyses of agreement between double-leg and single-leg weight-bearing radiographs according to fracture history and osteotomy status in operated limbs.

Group	Predefined tolerance	*n*	Mean bias (DL − SL)	Mean absolute error	% within tolerance	95% CI
*Lateral distal femoral angle (LDFA)*						
Fracture	3°	29	−0.662°	2.020°	79.3%	[61.6–90.2%]
Non fracture	3°	35	+0.891°	1.805°	85.7%	[70.6–93.7%]
Osteotomy	3°	26	+0.962°	2.372°	76.9%	[57.9–89.0%]
Non osteotomy	3°	38	−0.343°	1.581°	86.8%	[72.7–94.2%]
*Medial proximal tibial angle (MPTA)*						
Fracture	3°	29	−0.137°	1.970°	86.2%	[69.4–94.5%]
Non fracture	3°	35	+0.161°	1.712°	88.6%	[74.0–95.5%]
Osteotomy	3°	26	−0.579°	1.575°	92.3%	[75.9–97.9%]
Non osteotomy	3°	38	+0.440°	2.002°	84.2%	[69.6–92.6%]
*Joint line convergence angle (JLCA)*						
Fracture	2°	29	+0.389°	1.262°	79.3%	[61.6–90.2%]
Non fracture	2°	35	+0.021°	0.975°	82.9%	[67.3–91.9%]
Osteotomy	2°	26	+0.138°	1.154°	76.9%	[57.9–89.0%]
Non osteotomy	2°	38	+0.222°	1.072°	84.2%	[69.6–92.6%]

**Table 4:** Bland–Altman agreement between double-leg and single-leg measurements in 91 limb-encounters. Bias is DL − SL; LoA, limits of agreement. Confidence intervals were obtained by resampling patients with replacement for 10,000 iterations while retaining all observations contributed by each sampled patient.

Parameter	*n*	Bias, ° (cluster 95% CI)	Lower LoA, ° (cluster 95% CI)	Upper LoA, ° (cluster 95% CI)
LPFA	91	−0.92 (−2.58 to 0.45)	−12.77 (−19.62 to −7.47)	10.93 (6.23 to 15.46)
LDFA	91	0.00 (−0.60 to 0.51)	−5.46 (−6.91 to −3.69)	5.46 (3.40 to 7.24)
MPTA	91	−0.06 (−0.45 to 0.35)	−4.26 (−5.35 to −3.27)	4.15 (3.08 to 5.31)
LDTA	91	0.39 (−0.29 to 1.17)	−5.31 (−6.32 to −3.94)	6.09 (5.11 to 7.04)
JLCA	91	0.13 (−0.15 to 0.46)	−2.74 (−3.21 to −2.19)	3.00 (2.22 to 3.84)

## Discussion

Accurate assessment of lower extremity coronal alignment is fundamental to surgical planning and postoperative evaluation, yet the optimal weight-bearing condition for radiographic assessment remains uncertain. In this study, SL and DL weight-bearing radiographs demonstrated 69.2% overall agreement within predefined angular tolerance thresholds, with minimal mean bias across all measured parameters. Despite the small average differences, nearly one-third of limbs demonstrated at least one measurement outside the predefined tolerance threshold, indicating that agreement between SL and DL radiographs is not uniform across all patients.

Previous studies have primarily evaluated agreement between SL and DL radiographs in patients with knee osteoarthritis or elective deformity correction. The present Bland–Altman analysis demonstrates why group-level mean differences alone are insufficient: average biases were close to zero, yet the 95% LoA spanned approximately 23.7° for LPFA, 10.9° for LDFA, 8.4° for MPTA, 11.4° for LDTA, and 5.7° for JLCA. Furthermore, the proportional-bias analysis indicates that JLCA disagreement increased at larger JLCA values. These findings show that meaningful individual differences may occur without a large overall mean bias [3, 15, 16].

Unlike prior studies, this investigation evaluated patients following lower extremity trauma and reconstructive surgery. Agreement was substantially lower in operated limbs than in non-operated limbs, suggesting that prior surgical intervention or underlying pathology may increase variability under different loading conditions. Altered joint mechanics, retained implants, postoperative pain, and soft-tissue imbalance have all been shown to influence lower-extremity alignment under weight-bearing conditions [9, 11]. These factors may explain why operated limbs demonstrated greater variability between SL and DL radiographs.

Agreement also differed among individual alignment parameters. MPTA demonstrated the highest agreement, whereas LDFA and JLCA were more likely to exceed predefined tolerance thresholds. These findings are consistent with previous work demonstrating that tibial alignment is generally more reproducible than measurements involving femoral orientation or joint line geometry, which are more sensitive to limb rotation and patient positioning [10, 12, 13]. Similarly, Brouwer et al. demonstrated that differences in patient positioning alone can significantly influence measured mechanical alignment, emphasizing the importance of weight-bearing conditions during radiographic assessment [14].

When discrepancies occurred, they were most commonly isolated to a single alignment parameter rather than representing combined femoral and tibial deformity. This observation suggests that localized changes in alignment or soft-tissue balance may account for many of the differences observed across imaging conditions, rather than global changes in limb alignment [9, 11]. In particular, JLCA demonstrated relatively frequent measurements outside tolerance despite low mean absolute error, likely reflecting both its narrower tolerance threshold and the influence of intra-articular and ligamentous factors under physiologic loading [3, 11].

Exploratory subgroup analyses demonstrated no association between fracture history and disagreement between SL and DL radiographs. In contrast, limbs with a history of osteotomy demonstrated a higher proportion of discrepant measurements, particularly for LDFA. However, these differences were not statistically significant and should be considered hypothesis-generating because of the limited sample size. Larger prospective studies are needed to determine whether specific reconstructive procedures increase sensitivity to weight-bearing conditions.

Several limitations should be considered. First, the retrospective design and exclusion of encounters without paired SL–DL images introduce selection bias. Second, subgroup analyses were limited by sample size. Third, measurements were obtained at one institution. Fourth, the prespecified 3° and 2° thresholds are descriptive and have not been validated as minimal clinically important differences for comparing these imaging conditions; sensitivity analyses showed that the absolute agreement percentages varied with the selected threshold. Fifth, sagittal knee alignment, including fixed flexion deformity and genu recurvatum, was not measured or used as an exclusion criterion. Sixth, exact positioning and quantitative SL loading were not recorded retrospectively. Seventh, although patient-cluster bootstrap methods accounted for patients contributing multiple observations, the analysis remains based on a modest 32-patient cohort. Finally, although two blinded evaluators independently measured each parameter and their mean was used for analysis, the individual evaluator-level values were not retained after averaging. Interobserver ICCs therefore could not be calculated retrospectively, and the contribution of observer variability to the observed SL–DL differences could not be quantified [17].

SL and DL weight-bearing radiographs showed small average differences. Still, nearly one-third of limbs exceeded at least one prespecified descriptive threshold, particularly among operated extremities and for LDFA and JLCA. This study compared agreement and did not evaluate accuracy against an external reference standard. Accordingly, SL imaging should be considered potentially complementary to DL imaging in selected postoperative patients, especially when a measurement lies near a clinical decision threshold; these findings do not establish that SL radiographs are superior or more accurate.

## Data Availability

The datasets generated and/or analyzed during the current study are not publicly available due to institutional policies regarding patient data but are available from the corresponding author on reasonable request.
